# Patterns of bacterial pathogens and their antimicrobial susceptibility from blood culture specimens in Wad Medani, Sudan: a four-year laboratory-based, cross-sectional study

**DOI:** 10.4314/ahs.v25i1.3

**Published:** 2025-03

**Authors:** Yousif B Hamadalneel, Hifa O Ahmed, Marwa F Alamin

**Affiliations:** 1 Department of Clinical Pharmacy & Pharmacy Practice, Faculty of Pharmacy, University of Gezira, Wad Medani, Sudan; 2 Department of Molecular Biology, Institute of Endemic Disease, Khartoum University, Khartoum, Sudan

**Keywords:** patterns, bacterial pathogens, antimicrobial susceptibility, blood culture, specimens, Wad Medani, Sudan

## Abstract

**Background:**

Antibiotic-resistant bacterial bloodstream infections are rapidly emerging, which makes successful treatment challenging. Therefore, this study aimed to determine the patterns of bacterial pathogens and their antimicrobial susceptibility from blood culture samples.

**Methods:**

This was a cross-sectional study. All clinical samples were collected from patients at Wad Medani and investigated at the Pathology Center for Diagnosis and Research, Faculty of Medicine, University of Gazira, Sudan, from the 1st of January, 2020, to the 15th of October, 2023.

**Results:**

Overall, 577 blood samples were cultured. Among these samples, 86 (14.9%) exhibited bacterial growth. S. aureus (40.7%) and E. coli (40.7%) were the most frequently isolated bacteria. The most sensitive drugs to S. aureus were vancomycin 100% (13/13) and linezolid 86.7% (13/15), whereas the most sensitive drugs to E. coli were norfloxacin 88.9% (8/9), imipenem 85.7% (6/7), and levofloxacin 84% (21/25). The rate of bacterial growth has steadily increased over time, from 5% in 2020 to 24.9% in 2023.

**Conclusions:**

This study revealed a modest rate of 14.9% of bloodstream infections, which has steadily increased over the years. The most frequently isolated bacteria were S. aureus and E. coli. Vancomycin was the most susceptible drug to isolated bacteria.

## Introduction

Bloodstream infections (BSIs) account for the most common cause of death worldwide^1^. The terms BSIs and bacteremia are frequently applied interchangeably once a microorganism grows from a blood culture that is collected from a patient with clinical signs of infection after contamination has been ruled out^2^. Antibiotic-resistant bacterial BSIs are rapidly emerging, especially in gram-negative bacteria, which makes successful treatment challenging^3^. The emergence and rapid spread of resistance to antibiotics can be attributed, as in several Sub-Saharan African countries, to outdated national guidelines for antibiotic use, inadequate laboratory facilities for performing blood cultures, and antimicrobial drug susceptibility tests^4^. Blood cultures are a crucial part of the assessment of a variety of diseases, especially in patients with suspected sepsis^5^. A solid understanding of empirical local antibiotic therapy on the basis of evidence is crucial for treating specific microorganisms, identifying antibiotic resistance patterns, and creating national and international research initiatives^6^.

However, identifying the offending pathogen and determining the sensitivity pattern of the isolates via bacterial culture remain the cornerstones for the definitive diagnosis and management of BSIs^7^. The most frequently isolated microorganisms in BSIs are bacteria such as Enterobacter spp., Streptococcus aureus, coagulase-negative Staphylococcus (CoNS), Klebsiella pneumoniae, and Escherichia coli (E. coli) and fungi such as Candida spp^8^,^9^.

The World Health Organization (WHO) global report on the surveillance of antimicrobial resistance revealed that there are commonly no local data on antibiotic resistance and an absence of data on the most prevalent pathogens^10^. Furthermore, a recent report on antimicrobial resistance in the WHO African Region revealed that most of the studies were from Ethiopia, and data concerning antimicrobial resistance in Sudan are lacking^11^. Therefore, baseline data that can offer essential guidance for antimicrobial treatment of specific pathogens and bridge the gaps that influence future collaboration and data sharing in regional as well as national surveillance projects should be developed. This study aimed to determine the patterns of bacterial pathogens and their antimicrobial susceptibility from blood culture samples in Wad Medani, Sudan.

## Methods

### Study Site

Wad Medani, Gezira State, Sudan.

### Study Design

This was a cross-sectional study.

### Sample size and data collection

All clinical samples were collected from patients at Wad Medani and investigated at the Pathology Center for Diagnosis and Research (PCDR), Faculty of Medicine, University of Gazira, Sudan, from the 1st of January, 2020, to the 15th of October, 2023; these patients were included in the study.

### Sample collection and laboratory methods

#### Sample collection

Clinical blood samples were collected from the study population using standard microbiological methods. Aseptic blood collection was used to obtain 10 mL of adult venous whole blood, 5 mL of pediatric blood, and 2 mL of neonatal blood^12^. For culture and drug sensitivity, every sample was subsequently sent aseptically to the PCDR microbiology laboratory.

#### Identification of the Isolated Organism

The collected blood samples were inoculated onto MacConkey agar and blood agar plates. Cultures were incubated in an aerobic atmosphere at 37°C for 24 hours. After 24 hours, all of the plates were first checked for growth, and those that showed no growth underwent additional incubation for up to 48 hours. For all positive cultures, morphological characteristics, Gram staining, and confirmatory biochemical tests were used to identify the bacterial isolates.

Gram-positive bacteria were identified via the catalase reaction, coagulase test, optochin test, bacitracin test, and hemolytic activity test on blood agar^12^. Additionally, gram-negative bacteria were identified by inoculation on MacConkey agar plates, followed by biochemical tests such as H2S production, indole production, utilization of citrate/carbohydrates, urease tests, and oxidase tests^12^.

#### Antimicrobial susceptibility

The antimicrobial susceptibilities of the bacterial isolates were ascertained by Mueller–Hinton agar plates (Oxoid, England) using the Kirby–Bauer disk diffusion method according to CLSI 2020 guidelines^13^.

Gram-positive isolates were tested against the following antimicrobials: vancomycin (30 µg), cotrimoxazole (1.25/23.75 µg), cefuroxime (30 µg), ampicillin/sulbactam (10/10 µg), imipenem (10 µg), ceftriaxone (30 µg), cefotaxime (30 µg), amoxicillin/clavulanic (20/10 µg), tetracycline (30 µg), ciprofloxacin (5 µg), gentamycin (10 µg), linezolid (30 µg), cloxacillin (5 µg), levofloxacin (5 µg), cephalexin (30 µg), roxithromycin (50 mg), lincomycin (5 µg), chloramphenicol (30 µg), ceftriaxone (30 µg), ofloxacin (5 µg), amikacin (30 µg), norfloxacin (10 µg), erythromycin (15 µg), and clindamycin (2 µg) (13). Gram-negative isolates were tested against ampicillin/sulbactam (10/10 µg), cotrimoxazole (1.25/23.75 µg), norfloxacin (10 µg), tetracycline (30 µg), cloxacillin (5 µg), gentamycin (10 µg), imipenem (10 µg), ampicillin (10 µg), amoxicillin/clavulanic (20/10 µg), ciprofloxacin (5 µg), nitrofurantoin (300 µg), meropenem (10 µg), levofloxacin (5 µg), meropenem (10 µg), chloramphenicol (30 µg), ceftriaxone (30 µg), nalidixic acid (30 µg), ofloxacin (5 µg), piperacillin/tazobactam (100/10 µg), erythromycin (15 µg), amikacin (30 µg), cefuroxime (30 µg), and cefotaxime (30 µg)^13^. The CLSI 2020 guideline breakpoints were used to interpret zone diameters^13^.

### Quality control

As standard practice throughout the whole laboratory work process, quality control procedures were put in place to ensure the validity of the results. Before usage, the normal shelf-life of the culture media, staining reagents, and antibiotic discs were examined^14^. All culture plates and antibiotic discs were prepared and autoclaved at 121 °C for 15 minutes, after which they were kept at the stated refrigeration temperature. The standard reference bacterial strains were examined as positive controls on agar plates with antibiotic discs and biochemical assays^14^. The samples were handled carefully by qualified microbiologists.

### Statistical analysis

The Statistical Package for Social Science (SPSS) version 27.0 was used to analyze the data. The means and standard deviations (SDs) were used to present the quantitative data, while the qualitative data were presented as frequencies (percentages).

## Results

### Sample general characteristics

**Figure 1:**
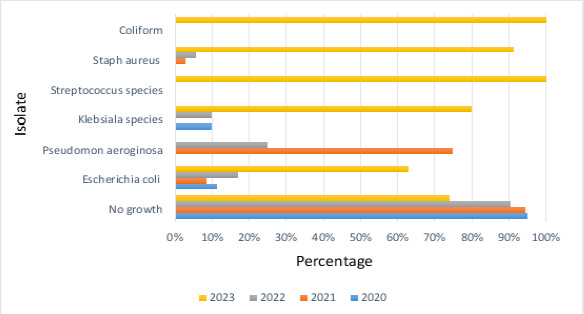
Percentages of bacterial isolates from the 1^st^ of January, 2020, to the 15^th^ of October, 2023

Over the four-year study period, 577 blood samples were cultured. Among these samples, 86 (14.9%) exhibited bacterial growth. Figure 1 shows the distribution of organisms found throughout this study period. The mean age of the patients included in the sample was 38.23 ± 24.31 [standard deviation (SD)] years, of whom 51 (8.8%) were less than one year. A total of 299 (51.8%) patients were males, and 278 (48.2%) were females.

### Bacterial profile

In total, our results revealed that 86 (14.9%) blood samples were positive for bacterial growth, 36 (41.86%) of which were gram-positive, whereas the majority 50 (58.14%) were gram-negative bacteria.

**Figure 2:**
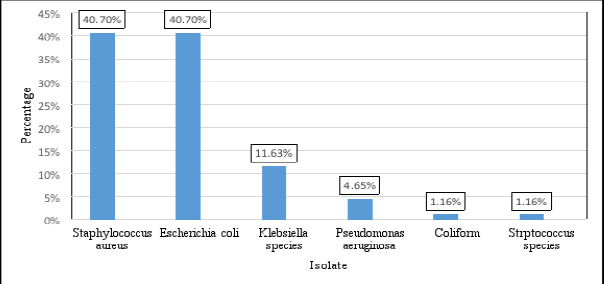
Bacterial isolates from blood samples

Overall, six types of bacteria were isolated. Staphylococcus aureus (S. aureus) and E. coli were the most frequently isolated bacteria, followed by Klebsiella spp. (species) and Pseudomonas aeruginosa (P. aeruginosa) (Figure 2). Our data revealed that S. aureus was the most abundant gram-positive bacteria, whereas E. coli was the most common gram-negative bacteria (Figure 2).

### Antimicrobial Susceptibility Profile

**Figure 3:**
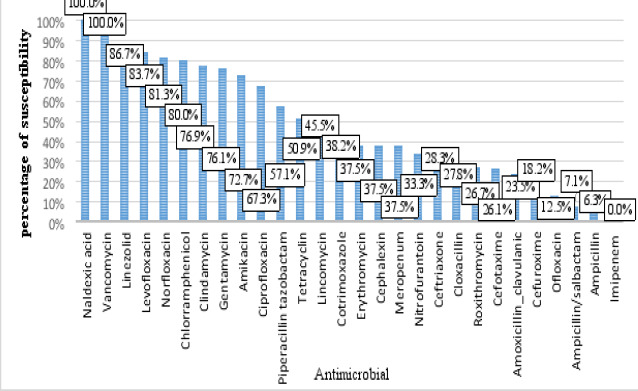
Patterns of antimicrobial susceptibility among bacteria detected in blood samples

The obtained data revealed that out of the 28 drugs tested. Vancomycin 100% (13/13) was the most susceptible drug to isolated bacteria, followed by linezolid 86.7% (13/15) and levofloxacin 83.7% (14/49). In contrast, imipenem showed total resistance (12/12) to the isolated bacteria (Figure 3). Furthermore, over time, many drugs have decreased susceptibility, whereas others have increased susceptibility, as presented in Table 1.

**Table 1:** Changes in antibiotic susceptibility over time

Antibiotic	2020	2021	2022	2023
Ampicillin/sulbactam	0	ND	33.3%	5%
Vancomycin	ND	ND	100%	100%
Cotrimoxazole	0%	57.1%	28.6%	62.5%
Cephalexin	ND	ND	ND	37.5%
Tetracycline	33.3%	28.6%	44.1%	57.9%
Cefotaxime	0	100%	0	17.2%
Ciprofloxacin	40%	ND	75%	70%
Levofloxacin	60%	ND	66.7%	87.8%
Linezolid	ND	ND	ND	86.7%
Cloxacillin	ND	ND	ND	27.8%
Roxithromycin	ND	ND	ND	26.7%
Lincomycin	ND	ND	ND	45.5%
Gentamycin	40%	ND	100%	79.5%
Piperacillin tazobactam	40%	ND	100%	50%
Chloramphenicol	100%	ND	50%	84.2%
Ofloxacin	40%	ND	0	0
Amikacin	66.7%	71.4%	87.5%	69.2%
Norfloxacin	66.7%	80%	66.7%	44.4%
Nitrofurantoin	100	ND	ND	33.3
Amoxicillin/clavulanic	ND	ND	ND	23.5%
Ampicillin	ND	21.7%	0	0
Cefuroxime	ND	14.3%	0	30%
Clindamycin	ND	ND	0	34.2
Ceftriaxone	0	ND	34.1%	34.4%
Erythromycin	ND	ND	50%	35.7%
Meropenem	ND	ND	ND	37.5%
Imipenem	ND	ND	ND	0
Nalidixic acid	ND	ND	ND	100%

ND: not done

Our data revealed that, collectively, vancomycin (13/13) was 100% sensitive to S. aureus bacteria, followed by gentamicin 93.3% (14/15), chloramphenicol 87.7% (13/15), levofloxacin 87.5% (14/16) and linezolid 86.7%(13/15), while isolated S. aureus was 100% (0/2) resistant to imipenem and ofloxacin. On the other hand, Streptococcus spp. were also collectively 100% (1/1) resistant to ceftriaxone, norfloxacin, ciprofloxacin, and levofloxacin, while showing complete sensitivity of 100% (1/1) to gentamicin and amikacin (Table 2).

**Table 2:** Sensitivity of gram-positive bacteria to different antimicrobial agents

Antibiotic	*S. aureus*	*Streptococcus species*	Overall
Ampicillin/sulbactam	7.1% (1/14)	ND	7.1%
Vancomycin	100% (13/13)	ND	100%
Cotrimoxazole	55% (11/20)	ND	55%
Cephalexin	37.5% (6/16)	ND	37.5%
Tetracycline	60.7% (17/28)	ND	60.7%
Cefotaxime	26.7% (4/15)	ND	26.7%
Ciprofloxacin	73.7% (14/19)	0 (0/1)	70%
Levofloxacin	87.5% (14/16)	0(0/1)	82.4%
Linezolid	86.7% (13/15)	ND	86.7%
Cloxacillin	33.3% (5/15)	ND	33.3%
Roxithromycin	26.7% (4/15)	ND	26.7%
Lincomycin	45.5% (5/11)	ND	45.5%
Gentamycin	93.3% (14/15)	100% (1/1)	93.8%
Chloramphenicol	86.7% (13/15)	ND	86.7%
Ofloxacin	0 (0/2)	ND	0%
Amikacin	66.7% (2/3)	100% (1/1)	75%
Norfloxacin	100% (2/2)	0 (0/1)	66.7%
Amoxicillin/clavulanic	66.7% (2/3)	0 (0/1)	50%
Cefuroxime	50% (2/4)	ND	50%
Clindamycin	76.9% (10/13)	ND	76.9%
Ceftriaxone	41.2% (7/17)	0 (0/1)	38.9%
Erythromycin	40% (6/15)	ND	40%
Imipenem	0(0/2)	ND	0

*S. aureus: Staphylococcus aureus;* ND: not done

Our data revealed that among the gram-negative bacteria, norfloxacin (88.9%, 8/9), imipenem 85.7% (6/7), and levofloxacin 84% (21/25) were the most sensitive drugs to E. coli (Table 3). Amikacin was collectively 100% (3/3) sensitive to P. aeruginosa, whereas chloramphenicol and imipenem were collectively 100% (2/2) sensitive to Klebsiella spp. (Table 3). On the other hand, levofloxacin, norfloxacin, meropenem, and imipenem were collectively 100% (1/1) sensitive to isolated coliform bacteria (97.7%) (Table 3).

**Table 3:** Sensitivity of gram-negative bacteria to different antimicrobial agents

Antibiotic	*E. coli*	*P. aeruginosa*	*Klebsiela*	*Coliform*	Overall
Ampicillin/sulbactam	9.1% (1/11)	ND	0 (0/3)	ND	7.1%
Cotrimoxazole	37.5% (9/24)	33.3% (1/3)	0% (0/6)	0 (0/1)	29.4%
Tetracycline	27.8% (5/18)	33.3% (1/3)	83.5% (5/6)	100% (1/1)	42.9%
Cefotaxime	20% (4/20)	66.7% (2/3)	14.3% (1/7)	ND	23.3%
Ciprofloxacin	69.6% (16/23)	ND	50% (3/6)	ND	65.5%
Levofloxacin	84% (21/25)	ND	83.3% (5/6)	100% (1/1)	84.4%
Cloxacillin	0(0/1)	ND	0(0/2)	ND	0
Gentamycin	60.9% (14/23)	ND	85.7(6/7)	ND	66.7%
Piperacillin tazobactam	54.5% (6/11)	ND	66.7% (2/3)	ND	57.1%
Chloramphenicol	62.5% (5/8)	ND	100% (2/2)	ND	70%
Ofloxacin	10% (1/10)	ND	25% (1/4)	ND	14.3%
Amikacin	72.4% (21/29)	100% (3/3)	57.1% (4/7)	ND	71.8%
Nitrofurantoin	0 (0/1)	ND	ND	0 (0/1)	0
Norfloxacin	88.9% (8/9)	ND	66.7% (2/3)	100% (1/1)	84.6%
Amoxicillin/clavulanic	22.2%(2/9)	ND	0 (0/3)	0 (0/1)	15.4%
Ampicillin	11.1% (1/9)	0% (0/3)	0% (0/3)	ND	6.7%
Cefuroxime	11.1% (1/9)	0% (0/3)	25% (1/4)	0% (0/1)	11.8%
Ceftriaxone	26.1% (6/23)	ND	0 (0/5)	ND	21.4%
Erythromycin	0 (0/1)	ND	ND	ND	0
Meropenem	20% (1/5)	ND	50%(1/2)	100%(1/1)	37.5%
Imipenem	85.7% (6/7)	ND	100%(2/2)	100%(1/1)	90%
Nalidixic acid	ND	ND	100(1/1)	ND	100%

*E. coli; Escherichia coli; P. aeruginosa; Pseudomonas aeruginosa*; ND; Not done

## Discussion

This study aimed to estimate the frequency of clinically important blood–borne pathogens and to examine their antimicrobial resistance patterns. The bacterial isolation rate in this study was 14.9%, which was comparable with the results conducted in Dhaka, Bangladesh 13.6%^15^, Germany 13.2%^16^, Ghana 13.1%^17^, and Kathmandu, Nepal 12.6%^18^. However, fewer than studies have been conducted in Kigali, Rwanda 31.7%^19^, Mekelle Hospital, Ethiopia 28%^20^, and Cairo University Children Hospital 31.7%^21^. However, lower rate have been reported in other study conducted at Arba Minch General Hospital, Ethiopia 9.8%^22^. This difference in the bacterial isolation rate from blood samples among countries could be due to variations in the technical facilities for laboratories, patient population, geographic location, etiological agent epidemiology, seasonal fluctuations, and differences in infection control regulations between countries.

In this study, the majority of the isolated bacteria were gram-negative bacteria. This finding was in line with studies conducted in Dhaka, Bangladesh, which reported that the majority were gram-negative 72.1%^15^, Kigali, Rwanda 68.3%^19^, Cairo University Children Hospital 65.3%^21^, and in Nigeria 67.6%^23^. In contrast, gram-positive bacteria have been reported as the most common bacteria in Mekelle, Ethiopia 72.2%^20^; Italy 57.8%^24^; and Arba Minch Hospital, Ethiopia 59.1%^22^. On the other hand, a study conducted in Lahore, Pakistan showed that the percentages of gram-positive and gram-negative bacteria were nearly the same^25^.

The most common type of bacteria isolated in this study was S. aureus, followed by E. coli, which contradicts other studies conducted in Germany reported that E. coli 25.4% was the most commonly detected pathogen, followed by S. aureus 15.2%^16^; Karbala, Iraq; E. coli 22.1%, S. aureus 20.3%^26^; and in Nigeria, E coli 29.4% and S. aureus 23.5%^23^. However, another study conducted in Dhaka, Bangladesh, identified Salmonella typhi as the most frequently isolated organism at 36.9%^15^, whereas Enterococcus spp. at 23.71% and Acinetobacter spp. at 22.16% were the most frequently isolated bacteria in India^27^.

Regarding the susceptibility profile of isolated bacteria. Overall, vancomycin, linezolid, and levofloxacin were the most susceptible drugs, whereas imipenem showed total resistance to isolated bacteria. The findings of this study differed from those of other national studies. In a study conducted at Mekelle Hospital, Ethiopia, gentamicin, ciprofloxacin, and amoxicillin-clavulanic acid were the most sensitive drugs to isolated bacteria, while a high resistance rate was reported for trimethoprim-sulphamethoxazole 70.1%, ofloxacin 62.5%, and ceftriaxone 58.9%^20^. However, a study carried out in Kigali, Rwanda, revealed high resistance rates to penicillin 91.8%, trimethoprim-sulfamethoxazole 83.3%, and ampicillin 81.8%, while bacteria exhibit high sensitivity to imipenem 98.1% and vancomycin 94.3%^19^. In addition, a study conducted in Nigeria revealed that all isolated bacteria were susceptible to meropenem and imipenem at 97.1%, whereas a low sensitivity rate of 38.2% for cotrimoxazole and 32.4% for ampicillin was reported^23^.

Our data revealed that S. aureus bacteria are collectively sensitive to vancomycin and highly sensitive to gentamicin, chloramphenicol, levofloxacin, and linezolid. The isolated S. aureus was completely resistant to imipenem and ofloxacin. This result was comparable with the results of most published studies. Of which, a study conducted at Mekelle Hospital, Ethiopia, which reported that all gram-positive bacteria are completely sensitive to vancomycin^20^, in Lahore, Pakistan, the sensitivity of gram-positive organisms to vancomycin, teicoplanin, and linezolid is 100%^25^, in Cairo University Children Hospital, gram-positive bacteria are 100% sensitive to vancomycin and linezolid, whereas all gram-positive bacteria are resistant to ciprofloxacin, cephalosporin, imipenem, and beta lactamase combinations as well as a high resistance rate to gentamicin, and levofloxacin^21^, in Nigeria all isolated gram-positive bacteria are sensitive to vancomycin^23^, and in Sharif Medical City Hospital, Pakistan, all gram-positive bacteria are 100% susceptible to vancomycin and linezolid^28^.

Among the gram-negative bacteria, norfloxacin, imipenem, and levofloxacin were the most sensitive drugs to E. coli; however, E. coli showed total resistance to cloxacillin, nitrofurantoin, and erythromycin as well as high resistance rates to ampicillin, cefuroxime, and ampicillin/sulbactam. This result contrasts with that of a study conducted in Lahore, Pakistan, which revealed that the most susceptible to gram-negative bacteria were colistin, imipenem, meropenem, and amikacin^25^. Furthermore, a study conducted in Karbala, Iraq, reported that the most sensitive drugs for E. coli were colistin 97%, imipenem 89%, meropenem 88%, and amikacin 79%, whereas the highest resistance rates were benzyl penicillin 93%, and oxacillin 86% (26).

P. aeruginosa is the greatest challenge pathogen because of its high prevalence of antibiotic resistance. In this study, P. aeruginosa exhibited total sensitivity to amikacin, in contrast to the results of a study conducted at Cairo University Children's Hospital, which reported total susceptibility to polymyxin and 50% susceptibility to amikacin^21^.

Another essential aspect is that the rate of bacterial growth steadily increased over time, from 5% in 2020 to 24.9% in 2023. This alarming increase in growth emphasizes the importance of continuous evaluation of bacterial BSI profiles and their antibiotic resistance patterns.

Integrating these results allows for the planning of long- and short-term strategies, and a good understanding of these trends would assist us in avoiding the use of highly ineffective empirical antibiotic choices, such as meropenem, ceftriaxone, and imipenem, which are currently being utilized.

## Strengths and limitations

In the strength of this study, include the analysis of data from four consecutive years, which provides an accurate depiction of the bacteriological profile and antibiotic resistance pattern of BSIs. Furthermore, the data were collected from the Pathology Center for Diagnosis and Research, Faculty of Medicine, University of Gazira. This facility works as the reference laboratory and encompasses all hospital settings at Wad Medani.

The current study has some limitations. In particular^1^, a complete patient profile was not available given the retrospective nature of the study, such as the patient setting, due to incomplete documentation^2^. Furthermore, there is no information available regarding the patients' diagnosis or medications administered, given that the clinical samples were obtained for diagnostic purposes independently of this study^3^. In addition to the absence of facilities in the microbiology laboratory, anaerobic microorganisms could not be included.

## Conclusions

This study revealed a modest rate of BSIs of 14.9%, which steadily increased over time, from 5% in 2020 to 24.9% in 2023. The majority of the isolated bacteria were gram-negative bacteria 58.14%. S. aureus and E. coli were the most frequently isolated bacteria. Vancomycin, linezolid, and levofloxacin were the most susceptible drugs to isolated bacteria, whereas imipenem showed total resistance. In the long term, these data can support related and subsequent studies in meta-analyses to further local, regional, and international guidelines.

## Data Availability

The datasets used and/or analyzed during the current study are available from the corresponding author upon reasonable request.
